# FTO variant is associated with changes in BMI, ghrelin, and brain function following bariatric surgery

**DOI:** 10.1172/jci.insight.175967

**Published:** 2024-08-01

**Authors:** Guanya Li, Yang Hu, Wenchao Zhang, Jia Wang, Lijuan Sun, Juan Yu, Peter Manza, Nora D. Volkow, Gang Ji, Gene-Jack Wang, Yi Zhang

**Affiliations:** 1 Center for Brain Imaging, School of Life Science and Technology, Xidian University and Engineering Research Center of Molecular and Neuroimaging, Ministry of Education, Xi’an, Shaanxi, China.; 2 International Joint Research Center for Advanced Medical Imaging and Intelligent Diagnosis and Treatment and Xi’an Key Laboratory of Intelligent Sensing and Regulation of Trans-Scale Life Information, School of Life Science and Technology, Xidian University, Xi’an, Shaanxi, China.; 3 Key Laboratory of Resource Biology and Biotechnology in Western China, Ministry of Education, School of Medicine, Northwest University, Xi’an, Shaanxi, China.; 4 Department of Digestive Surgery, Xijing Hospital, Air Force Medical University, Xi’an, Shaanxi, China.; 5 Laboratory of Neuroimaging, National Institute on Alcohol Abuse and Alcoholism, Bethesda, Maryland, USA.

**Keywords:** Neuroscience, Obesity

## Abstract

**BACKGROUND:**

A polymorphism in the fat mass and obesity-associated gene (FTO) is linked to enhanced neural sensitivity to food cues and attenuated ghrelin suppression. Risk allele carriers regain more weight than noncarriers after bariatric surgery. It remains unclear how FTO variation affects brain function and ghrelin following surgery.

**METHODS:**

Resting-state functional magnetic resonance imaging and cue-reactivity functional magnetic resonance imaging with high-/low-caloric food cues were performed before surgery and at 1, 6, and 12 months after surgery to examine brain function in 16 carriers with 1 copy of the rs9939609 A allele (AT) and 26 noncarriers (TT). Behavioral assessments up to 5 years after surgery were also conducted.

**RESULTS:**

The AT group relative to the TT group had smaller BMI loss at 12–60 months after surgery and lower resting-state activity in posterior cingulate cortex following laparoscopic sleeve gastrectomy (group-by-time interaction effects). Meanwhile, the AT group relative to the TT group showed greater food cue responses in dorsolateral prefrontal cortex (DLPFC), dorsomedial prefrontal cortex (DMPFC), and insula (group effects). There were negative associations of weight loss with ghrelin and greater activation in DLPFC, DMPFC and insula in the AT but not the TT group.

**CONCLUSION:**

These findings indicate that FTO variation is associated with the evolution of ghrelin signaling and brain function after bariatric surgery, which might hinder weight loss.

**TRIAL REGISTRATION:**

Chinese Clinical Trial Registry Center, ChiCTR-OOB-15006346.

**FUNDING:**

This work was supported by the National Natural Science Foundation of China (grant nos. 82172023, 82202252, 82302292); National Key R&D Program of China (no. 2022YFC3500603); Natural Science Basic Research Program of Shaanxi (grant nos. 2022JC-44, 2022JQ-622, 2023-JC-QN-0922, 2023-ZDLSF-07); Fundamental Research Funds for the Central Universities (grant nos. ZYTS23188, XJSJ23190, XJS221201, QTZX23093); and the Intramural Research Program of the National Institute on Alcoholism and Alcohol Abuse (grant no. Y1AA3009).

## Abstract 2

FTO variation is associated with the evolution of ghrelin and brain function after bariatric surgery, which might underlie lower weight-loss.

## Introduction

Obesity results from the imbalance between energy intake and expenditure combined with genetic susceptibility (1). The fat mass and obesity-associated gene (FTO) is a well-replicated gene locus of obesity across different ages and populations. A cluster of single nucleotide polymorphisms (SNPs) within intron 1 of FTO have been consistently associated with higher BMI and increased food intake (2). The rs9939609 A allele is one of the strongest risk factors for polygenic obesity, and this variant has population frequencies of 46% in Western and Central Europeans, and 16% in Chinese individuals (3). Carriers of two copies of the risk-conferring variant had a 1.67-fold increased risk of obesity relative with noncarriers (4). Although the rs9939609 A allele frequency is lower in Chinese populations compared with European populations, the FTO SNPs are strongly associated with obesity risk in the Chinese population (5).

FTO encodes an RNA adenosine demethylase that catalyzes the oxidative demethylation of N6-methyladenosine (m^6^A) (6, 7). Considerable epigenetic research showed that FTO-mediated m^6^A demethylation modulates the expression of lipid-related genes to regulate lipid metabolism (7). Furthermore, FTO has highest expression in the brain in humans, particularly in the cerebral cortex (4). Allelic variants in FTO modulate brain responses to food cues in regions involved in satiety (hypothalamus), food reward (ventral tegmental area [VTA] and nucleus accumbens), and inhibitory control of eating (prefrontal cortex), thereby enhancing neural sensitivity to food stimulation and increasing food intake (8, 9). In addition, FTO variants are linked to higher plasma levels of the hunger hormone ghrelin. Individuals homozygous for the rs9939609 A allele display an attenuated postprandial suppression of orexigenic hormone acyl-ghrelin compared with individuals homozygous for the low-risk T allele (10), which predisposes FTO variant carriers to higher energy intake and, consequently, higher fat mass (10).

The FTO genotype has also been associated with individual variability in weight loss in response to multiple obesity treatment methods, including diet, lifestyle interventions, physical exercise, and bariatric surgery (11–14). Laparoscopic sleeve gastrectomy (LSG), one of the most effective bariatric surgical procedures, produces sustained weight loss and reduces craving for high-calorie food after surgery (15). However, there is also a lower proportion of weight loss in individuals carried the rs9939609 A allele, with a greater and earlier weight regain 2 years after LSG in European population (16, 17). There might be significant effects of the rs9939609 genetic variant on weight loss in Chinese populations due to the similar effects of the FTO variant on obesity in both Western and Chinese populations (5), but the long-term evaluation is still lacking. At the same time, a better understanding of the modification effects of genetic variation on weight loss in response to LSG may help to develop more effective individualized strategies for obesity treatment.

Therapeutic benefits of LSG are partly mediated through its actions on the brain. The reduced appetite seen after surgery has been attributed to changes in functional and structural frontal-mesolimbic circuitry that regulate appetite, reward, and incentive motivation (18). After LSG, participants with obesity showed significant increased gray matter density/volume in the caudate, insula, hippocampus, amygdala, anterior cingulate cortex (ACC), and posterior cingulate cortex (PCC) and decreased activation in response to food cues in the striatum, VTA, and dorsolateral prefrontal cortex (DLPFC), which correlated with reduced food craving and weight loss (19). It remains unclear how the FTO gene polymorphism affects LSG-induced changes in brain function. It is possible that individuals carried the high-risk allele would retain high neural sensitivity to food cues after LSG, resulting in smaller weight loss and greater weight regain.

LSG involves removal of the gastric fundus where ghrelin is mainly produced. There were significant decreases in fasting plasma ghrelin levels after LSG, which were associated with less craving for high-calorie food cues and reduced DLPFC activation (19, 20) along with strengthened functional and structural connectivity with ventral ACC, a region important for self-control and executive functions (21, 22). Ghrelin was also associated with hippocampal function through its modulation of connectivity with the insula (23). These studies indicate that changes in ghrelin play an important role in regulation of energy homeostasis and weight loss following surgery in part through its actions in limbic, interoceptive, executive, and saliency brain regions (18). FTO overexpression reduced ghrelin mRNA m^6^A methylation, concomitantly increasing ghrelin mRNA and peptide levels (10), which implies that FTO variants may also have an effect on the evolution of ghrelin levels following surgery.

Here, we employed resting-state functional magnetic resonance imaging (RS-fMRI) to examine the amplitude of low-frequency fluctuations (ALFF), a measurement of the spontaneous fluctuation in blood oxygen level–dependent fMRI (BOLD-fMRI) signal intensity that has been investigated as a reliable biomarker for many neurological conditions and obesity (24). The cue-reactivity fMRI task with high caloric (HiCal) and low caloric (LoCal) food cues was also performed to evaluate the effect of the rs9939609 A allele on brain function following LSG. Functional connectivity analyses were performed to assess whole-brain effects of the FTO variant. Fasting blood samples were collected to measure the plasma concentrations of total ghrelin as well as other hormones involved in appetite control and metabolism, including GLP-1, glucagon, leptin, and insulin. We also conducted BMI measurements up to 5 years after surgery. We hypothesized that FTO variant carriers compared with noncarriers would have higher ghrelin levels and neural activation in regions involved with reward and inhibitory control of eating following surgery and that these factors would be associated with long-term outcomes of bariatric surgery.

## Results

### Participant characteristics.

**Figure 1:**
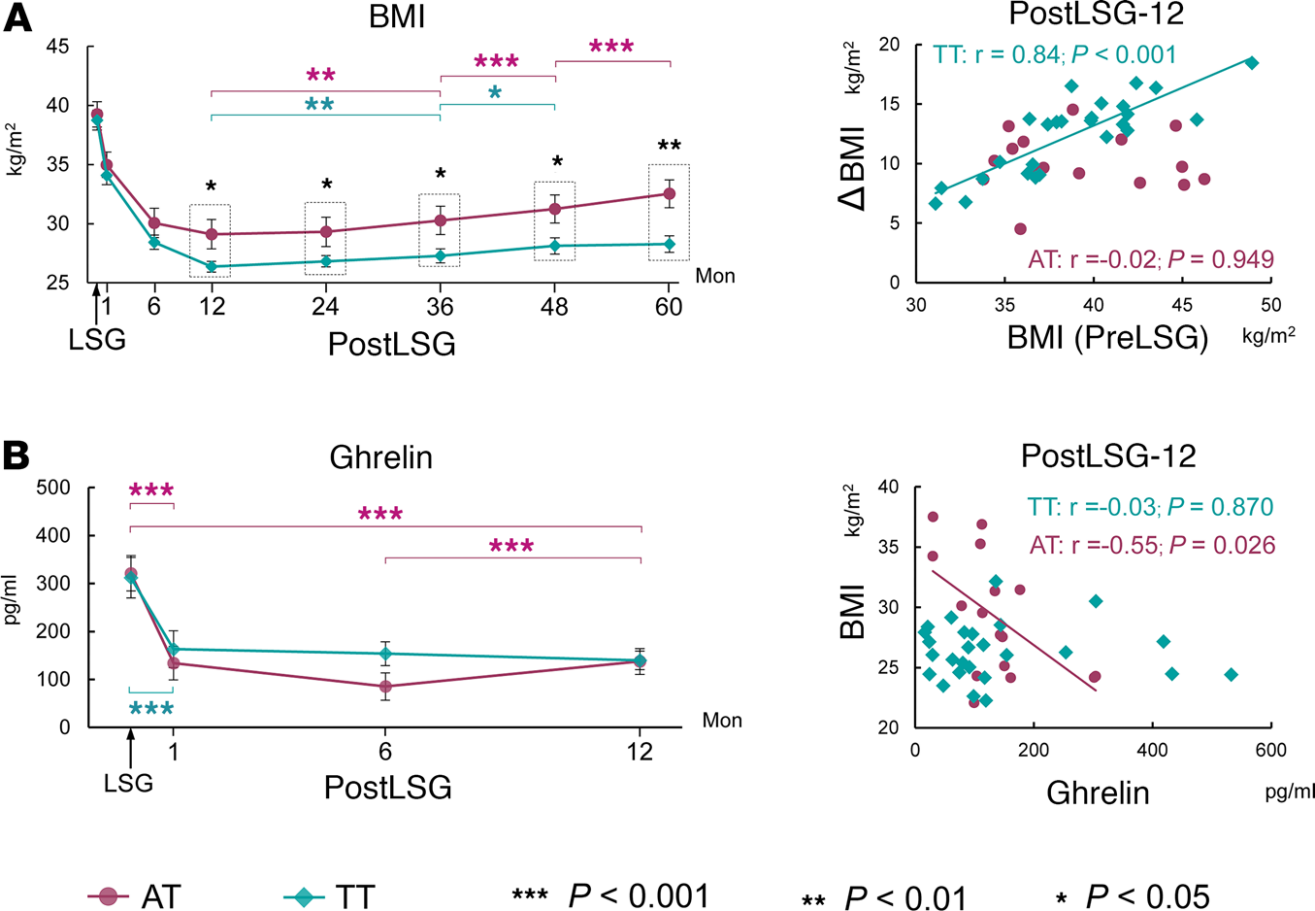
ANOVA showed significant interaction (group × time) effects on BMI and time effects on plasma ghrelin. (**A**) In the TT group, basal BMI was significantly correlated with BMI reductions at 12 months after LSG. (**B**) In the AT group, ghrelin plasma was increased at PostLSG-12, and levels were negatively correlated with BMI at 12 months after LSG.

Forty-two patients completed MRI assessments before surgery (PreLSG) and at 1, 6, and 12 months (PostLSG-1, -6, and -12) after surgery. All participants were also followed up at 24, 36, 48, and 60 months (PostLSG-24, -36, -48, and -60) after surgery and reported their BMI. Sixteen carriers with 1 copy of the rs9939609 A allele were classified as the AT group and 26 noncarriers were classified as the TT group. The rs9939609 A allele frequency of the current study population was 19.05%, which was 38.10% for the heterozygote and 0% homozygote of the A allele. A designated clinician rated anxiety and depression using the Hamilton Anxiety Rating Scale (HAMA) and the Hamilton Depression Rating Scale (HAMD). Participants completed the Yale Food Addiction Scale (YFAS) evaluation to assess addictive eating behaviors. At baseline, there were no significant differences in age, sex, BMI, HiCal and LoCal food craving, and scores on the YFAS, HAMD, and HAMA questionnaires between AT and TT groups (Table 1 and Figure 1).

ANOVA showed significant group × time interaction effects on weight (*F* = 4.91, *P* < 0.001) and BMI (*F* = 5.25, *P* < 0.001) and the percentage of excess BMI loss (EBMIL) (*F* = 5.17, *P* < 0.001). Post hoc tests showed that the TT group relative to the AT group had greater EBMIL (*t* = 2.26, *P* = 0.029, Cohen’s *d* = 0.72) and lower BMI (*t* = –2.44, *P* = 0.019, Cohen’s *d* = 0.77) at 12 months after LSG. Both the AT and TT groups showed significant weight regain starting 36 months after surgery; however, there was lower EBMIL in the AT group (Figure 1A). In the TT group, basal BMI was positively correlated with reduced BMI at 12 months after LSG (*r* = 0.84, *P* < 0.001) (Figure 1A) as well as 24, 36, 48, and 60 months following surgery (Supplemental Figure 1; supplemental material available online with this article; https://doi.org/10.1172/jci.insight.175967DS1), such that the higher the baseline BMI the greater the weight reduction. Meanwhile, the correlations between basal BMI and BMI reductions following LSG in the AT group were not significant (Figure 1A). In addition, we also evaluated the association between other previously reported single nucleotide polymorphisms (SNPs) of FTO gene (rs8050136, rs17817449, rs1421085, and rs1121980) and Mc4-R gene (rs17782313 and rs12970134) and the BMI loss following LSG. There were similar higher BMIs in FTO rs8050136, rs17817449, rs1421085, and rs1121980 variant carriers at 12 months after LSG due to the tight linkage disequilibrium with the FTO rs9939609 variant (Supplemental Tables 1 and 2). However, MC4-R variant carriers did not show group difference in BMI with noncarriers (Supplemental Table 2).

The interaction effect was not significant, but there were significant time effects on waist circumference (*F* = 202.08, *P* < 0.001), glucose (*F* = 26.23, *P* < 0.001), HiCal food craving (*F* = 12.63, *P* < 0.001), YFAS (*F* = 25.19, *P* < 0.001), HAMA (*F* = 18.05, *P* < 0.001), and HAMD (*F* = 8.30, *P* < 0.001) (Supplemental Table 3). There were significantly positive correlations between changes in HAMD and BMI loss at PostLSG-12 in the TT group (*r* = 0.42, *P* = 0.021) but not in the AT group (*r* = –0.22, *P* = 0.423). In the AT group, there were negative correlations between HiCal food craving at PostLSG-12 and EBMIL at 12, 24, 36, 48, and 60 months after LSG in the AT group (Supplemental Figure 2).

### Peripheral hormone measurements.

At baseline, there were no significant differences between the AT and TT groups in hormone measurements. LSG significantly decreased fasting plasma ghrelin (*F* = 37.97, *P* < 0.001), insulin (*F* = 14.81, *P* < 0.001), and leptin levels (*F* = 61.95, *P* < 0.001), but there were no significant group differences between the AT and TT groups. (Figure 1B). There were no significant time effects or group effects on glucagon and GLP-1 levels (Supplemental Tables 3 and 4). In the AT group, basal plasma ghrelin levels were negative correlated with BMI loss at PostLSG-6 (*r* = –0.52, *P* = 0.038) and PostLSG-12 (*r* = –0.57, *P* = 0.020). After LSG, ghrelin plasma in the AT group was increased at PostLSG-12 (*t* = 4.92, *P* < 0.001, Cohen’s *d* = 1.23) and levels were negatively correlated with BMI at 12 months (*r* = –0.55, *P* = 0.026) after LSG (Figure 1B) as well as 24, 36, 48, and 60 months following surgery (Supplemental Figure 3).

### Resting-state brain activity.

**Figure 2:**
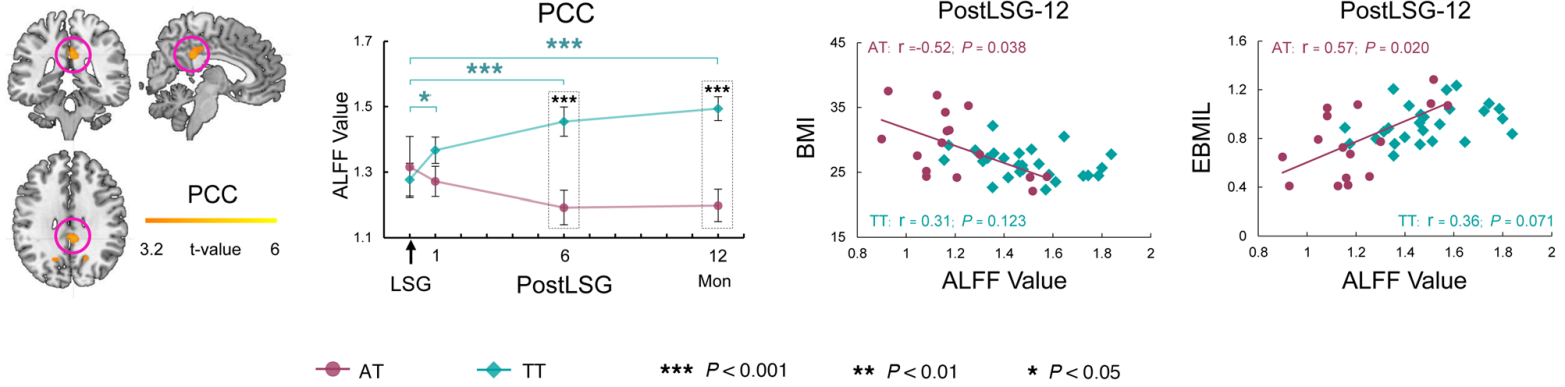
ANOVA showed significant interaction (group × time) effects on resting-state brain activity in PCC. PCC activity increased following LSG in the TT group at PostLSG-1, -6, and -12. Conversely, the AT group showed decreased ALFF in the PCC at PostLSG-12. BMI and the percentage of excess BMI loss (EBMIL) at 12 months after surgery in the AT group were correlated with PCC activity at PostLSG-12.

ALFF analysis was carried out to measure spontaneous fluctuations in resting-state BOLD fMRI signal intensity. ANOVA showed significant interaction effects of group on ALFF in PCC (Table 2 and Figure 2). Specifically, LSG increased PCC activity in the TT group at PostLSG-1, -6, and -12. Conversely, the AT group showed decreased ALFF in the PCC at PostLSG-12, which was significantly lower than that in the TT group (*t* = –4.897, *P* < 0.001, Cohen’s *d* = 1.56). The percentage of EBMIL in the AT group was negatively correlated with PCC activity at PostLSG-12 (*r* = –0.52, *P* = 0.038) (Figure 2). There were significant (*P* < 0.001) group effects on ALFF in orbitofrontal cortex (OFC), ACC, and PCC. The AT group showed lower activities in PCC and higher activities in OFC and ACC (Table 2). There were also significant (*P* < 0.001) time effects on ALFF in VTA, hippocampus/amygdala, and precentral/postcentral gyrus (PreCen/PostCen). Post hoc tests showed that LSG reduced VTA and hippocampus/amygdala activities and increased PreCen/PostCen activities (Table 2).

### Brain responses to food cues.

**Figure 3:**
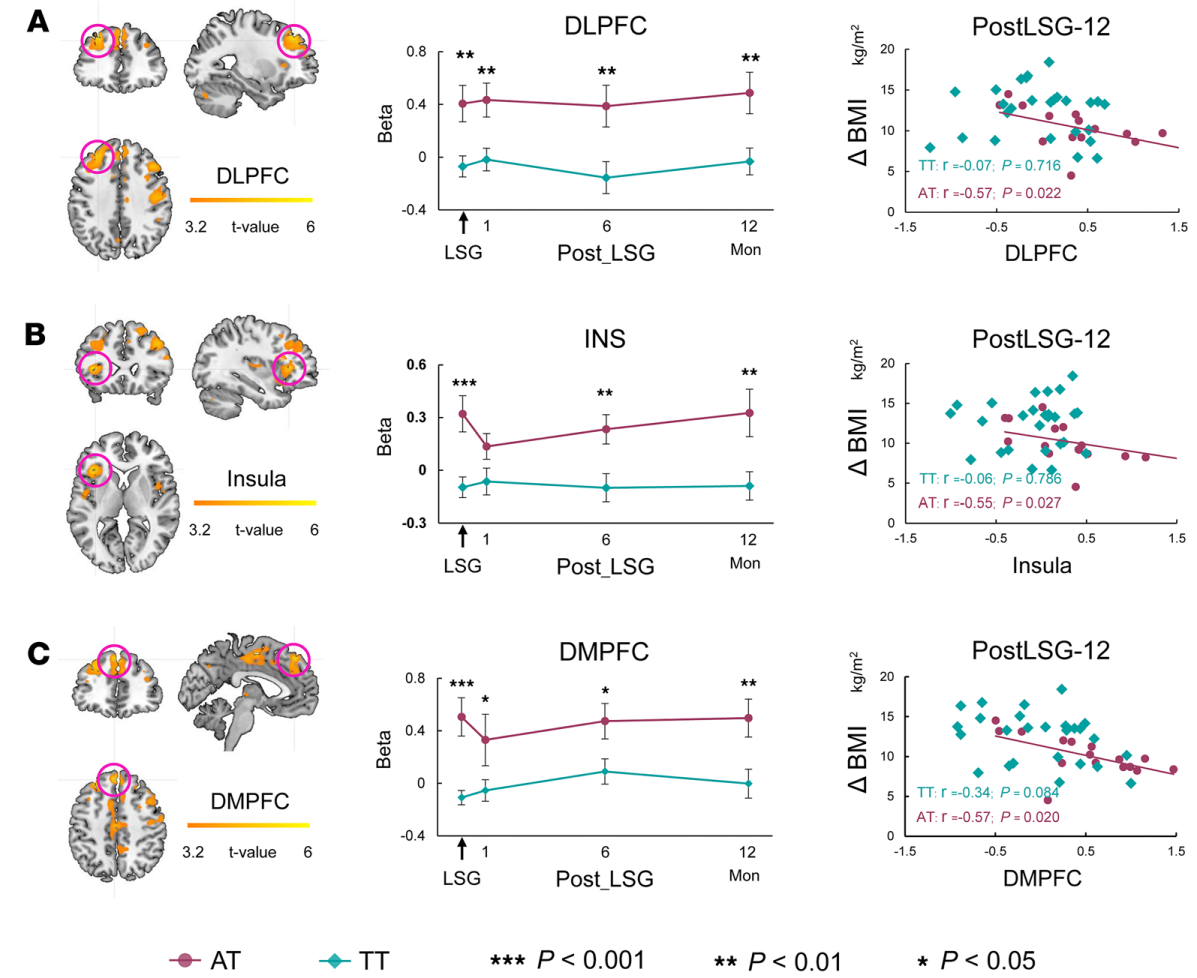
ANOVA showed group effects on brain responses to HiCal versus LoCal food cues. BMI reductions in the AT group were negatively correlated with food cue–induced activation in DLPFC, insula, and DMPFC at PostLSG-12.

The general-linear model, including HiCal and LoCal food cue condition regressors, was constructed to evaluate how the brain responded to HiCal versus LoCal food cues. There were no significant time or interaction effects, but there were significant (*P* < 0.001) group effects on brain responses to HiCal versus LoCal food cues (Table 2). The AT compared with the TT group showed greater activation before and after surgery in the DLPFC, dorsomedial prefrontal cortex (DMPFC), insula, supplemental motor area (SMA), and postcentral gyrus (*P*_FWE_ < 0.05, cluster-level correction) (Table 2 and Figure 3). BMI reductions in the AT group were negatively correlated with food cue–induced activation in DLPFC (*r* = –0.56, *P* = 0.022), insula (*r* = –0.55, *P* = 0.027), and DMPFC (*r* = –0.57, *P* = 0.021) at PostLSG-12 (Figure 3).

### Resting-state functional connectivity and PPI connectivity.

There were no significant interaction effects on the resting state functional connectivity; however, there were significant group effects for the brain connectivity of PCC-DLPFC and PCC–angular gyrus. The post hoc tests showed that the AT group relative to the TT group has lower functional connectivity before and after surgery (Supplemental Table 3 and Supplemental Figure 4). There were significant group effects for the psychophysiological interaction connectivity (PPI) connectivity of DLPFC-hippocampus and DMPFC-caudate in response to HiCal versus LoCal food cues. The post hoc tests showed that the AT group relative to the TT group has greater PPI connectivity before and after surgery (Supplemental Table 5 and Supplemental Figure 4).

## Discussion

Here we found that, compared with noncarriers, carriers of the rs9939609 A allele had less weight loss, lower resting-state activity in the PCC, and greater activation in the DLPFC, DMPFC, insula, SMA and postcentral gyrus following LSG. There were significant associations of weight loss with ghrelin, brain function, and food craving in the AT group, but none of these were significant in the TT group.

At baseline, there were no significant differences in BMI, food craving, and scores on the YFAS, HAMD, and HAMA questionnaires between the AT and TT groups. Individuals with obesity showed higher food craving and anxiety and depression levels compared with those of healthy-weight individuals. Both the AT and TT groups were at a similar raised weight status, which might prevent us from seeing difference by FTO genotype. After LSG, there was rapid weight loss in the first few months after surgery, likely due to purely restrictive and malabsorption mechanisms. Therefore, effects of rs9939609 FTO gene polymorphism on the magnitude of weight loss after LSG in the few months may not be noticeable (25). However, 36 months after surgery, A allele carriers of the rs9939609 FTO variant showed a lower proportion of surgery success and greater and earlier weight regain in Western populations (17). Our data showed that both the TT and AT groups achieved maximum weight loss at 12 months after LSG, and that the TT relative to the AT group had greater EBMIL and lower BMI, which indicated that FTO SNPs also play a vital role in weight change following LSG in Chinese populations. In the TT group, there was a significant positive correlation between baseline BMI and BMI loss at 12 months after LSG. This is consistent with results from previous reports showing that weight loss response was influenced by preoperative BMI, because people with higher BMI have more weight to lose. However, there was no similar association between baseline BMI and the smaller BMI loss in the AT group. In other words, individuals with the rs9939609 A allele had an attenuated weight loss response following LSG, especially among those with the highest BMI. At the same time, the rs9939609 A allele also affected weight regain outcomes in the long-term follow-up.

The FTO gene is associated with food intake regulation, and several lines of evidence indicate that A allele carriers exhibit higher craving for HiCal food and greater hunger, even in healthy-weight individuals (26). Although current results did not show a significant group difference in food craving between the AT and TT groups, there were negative correlations between craving for HiCal food cue at PostLSG-12 and EBMIL in the AT group, but not in the TT group, which suggests an interaction between the FTO genotype and eating behavior that impacts weight loss outcomes after LSG. The genetic predisposition of A allele carriers affects surgical outcomes, particularly when the initial restrictive effect decreases, resulting in smaller weight loss and more weight regain. Concomitantly, there were earlier rebound increases in plasma ghrelin in the AT group and ghrelin levels were negatively correlated with BMI at PostLSG-12. Ghrelin is the only known peripheral hormone that has orexigenic properties, stimulating appetite and increasing short-term food intake (27). Previous studies revealed that A allele carriers had impaired reduction in appetite-stimulating ghrelin levels, which was associated with more HiCal food craving after consuming a meal (10). The earlier rebound in ghrelin levels in the AT group relative to the TT group might promote parallel rebounds in food craving following LSG. Paradoxically, individuals with obesity show lower plasma ghrelin concentrations than the general population, which represent a physiological adaptation to the positive energy balance associated with obesity (27). LSG significantly decreases ghrelin due to the removal of the gastric fundus where ghrelin is mainly produced (18). The association between rebounding ghrelin and BMI at PostLSG-12 indicates a new adaptation to BMI, and the rising ghrelin levels might led to more weight regain in the AT group.

FTO-associated obesity risk is linked to altered brain function at rest and in response to food (8, 9, 28, 29). Previous resting-state fMRI studies showed normal weight carriers of the rs9939609 A allele have greater default mode network and sensorimotor network connectivity in regions involved in visceral perception and reward processing (28). FTO variant carriers also have greater responsivity to food cues in the ventral striatum, putamen, and posterior fusiform gyrus, which indicated that FTO gene variants modulate the neural correlates of visual food perception and raise the food craving and obesity risk (8, 9, 30). Enhanced neural sensitivity to food cues was also observed in individuals with obesity because of repeated access to highly palatable food (31). LSG altered functional and structural frontal-mesolimbic circuitry, reduced food craving, and improved cognitive function (18, 32). Here, LSG reduced VTA and hippocampus/amygdala activities and increased PreCen/PostCen activities, consistent with previous reports showing altered function of reward processing and visceral perception following LSG (18, 32).

However, there were significant interaction effects on PCC activity along with a distinct time course of brain functional changes between the AT and TT groups. The PCC is a prominent hub implicated in self-referential processing, supporting adaptation of the self to the internal context and external environment (18, 32). A randomized neurofeedback study targeting the PCC showed that increased activity in PCC can result in suppression of food craving in individuals with overweight or obesity, suggesting that increased interoceptive awareness after neurofeedback may have improved processing of internal appetite signals (33). The findings in the TT group are consistent with previous studies showing that increased PCC activity and enhanced resting-state functional connectivity of PCC after bariatric surgery were associated with short-term BMI loss. The lower PCC activity in the AT group was also significantly negatively associated with BMI as well as EBMIL at PostLSG-12, suggesting an effect on long-term weight loss following surgery.

There were also greater food cue–induced activations in the DLPFC, DMPFC, insula, SMA, and postcentral gyrus in the AT group compared with the TT group. The AT group relative to the TT group also has greater PPI connectivity of DLPFC-hippocampus and DMPFC-caudate before and after surgery. These brain regions have been associated with the anticipation of the rewarding value of food (31, 34, 35), self-referential processing involved with diverse functions linked with food reward (36), homeostatic regulation of food intake (37), and executive control of motor behavior (38). Greater activities and connectivity in those regions following LSG as well as their inverse association with BMI loss in the AT group suggest that the residual neural sensitivity to food cues had a negative effect on weight loss for FTO variant carriers.

LSG also reduced anxiety and depression as evidenced by reduced scores in the HAMA and HAMD, respectively, in both groups of patients with obesity, which indicates a beneficial effect of LSG in mood. There were significant correlations between changes in HAMD score and BMI loss at PostLSG-12 in the TT group, but not the AT group, suggesting that improvements in mood might also contribute to weight loss in noncarriers. The lesser weight loss in the AT group is adverse to the mood improvements and might produce a null relationship with changes in HAMD scores. Previous studies demonstrate that depression enhances the effect of FTO variants on BMI, such that individuals with depression have an additional 2.2% increase in BMI for each rs9939609 risk allele (A) compared with psychiatrically healthy individuals (39). The depressive status might also play an important role in the effects of FTO variants on BMI loss. Therefore, there is a need of further research to investigate the interaction among the FTO gene, BMI loss, and depression decline following LSG.

There are limitations to our study. Owing to strict exclusion criteria and long-term follow up, our sample size was somewhat limited and was small for the correlation analysis. The lack of a control group is a limitation to investigate surgery effects of the LSG. We did not measure changes in FTO expression level following LSG and levels of vitamin D or ANGPTL4, which are new factors involved in the weight loss after bariatric surgery (13, 40, 41). We performed MRI scans on participants after 12 hours of overnight fasting; it would be worth a further fed-state investigation because of the possible suppression of ghrelin in FTO variants carries following surgery. Although all the individuals underwent the LSG before 2018, there were 2 participants in the TT group and 1 participant in the AT group who claimed that their weight regain was due to less exercise, partially affected by the COVID-19 pandemic.

In conclusion, the current study investigated the association between FTO variants and the evolution of body weight, ghrelin, and brain function following LSG. Results indicated the earlier rebound in ghrelin levels, unimproved basal brain activity, and the residual greater neural sensitivity to food cues in FTO variants carriers might underlie lower weight loss. These results may help develop more effective individualized strategies for weight loss.

## Methods

### Sex as biological variable.

Male and female participants were included in our study. Sex was not considered as a biological variable.

### Participants.

Patients with obesity were recruited and completed LSG surgery at Xijing Gastrointestinal Hospital, which is affiliated with the Air Force Medical University in Xi’an, China. Participants were excluded from the study if they reported psychiatric/neurological disease, inflammatory/intestinal disease, organ dysfunction, a history of intestinal surgery, or use of any current medication, including weight loss medications, sleeping pills, and psychotropic medicines that could affect the central nervous system. As a result, a total of 42 patients completed MRI assessments before surgery and 1, 6, and 12 months after surgery. All of the participants were also followed up at 24, 36, 48, 60 months after surgery and reported their BMI. Genomic DNA from patients was extracted from peripheral blood using the standard proteinase K salting out method (42). FTO rs9939609 allelic discrimination was performed with an ABI7900HT detector (Applied Biosystems) and identified by Genemapper 4.0 software (42). Sixteen carriers with 1 copy of the rs9939609 A allele were classified as the AT group and 26 noncarriers were classified as the TT group. No individual was homozygous for the rs9939609 A allele. We also evaluated the association between other previously reported SNPs of FTO gene (rs8050136, rs17817449, rs1421085, and rs1121980) (43) and Mc4-R gene (44) (rs17782313 and rs12970134) and the BMI loss following LSG.

### Experimental design.

All participants underwent 12-hour overnight fasting. Fasting blood samples were collected and MRI scans were performed between 9 am and 10 am. Plasma concentrations of total ghrelin, GLP-1, glucagon, leptin, and insulin were measured using a Bio-Plex 200 suspension array system (BIO-RAD, Inc.) (19, 23) (Supplemental Methods). A designated clinician rated anxiety and depression using the HAMA and HAMD (45, 46). In addition, participants were required to complete the YFAS evaluation to assess addictive eating behaviors (23). All clinical measurements, including height, weight, and waist circumference, were measured before surgery and 1, 6, and 12 months after surgery. Self-report anthropometrics were used at 24, 36, 48, and 60 months after surgery to collect heights and weights, and BMI was calculated by experimenters. Surgical procedures were performed by the same surgeon for all patients.

### fMRI food cue–reactivity task.

The food cue–reactivity task consisted of three HiCal and three LoCal food cue blocks presented in pseudorandom order (19). Each block lasted 30 seconds, during which 10 pictures were presented for 3 seconds each with no intertrial interval, and the picture of a specific kind of food was presented at most once. There were 30 seconds of rest between blocks. After the task, participants were instructed to rate their level of craving for HiCal/LoCal food using a visual analog scale (range 0–100) (19).

### MRI acquisition.

The experiment was performed on a 3 T Signa Excite HD scanner (GE). A standard head coil was used with foam padding to reduce head motion. Following localizers, a coronal T2-weighted sequence (TR = 5727 ms, TE = 93 ms, matrix size = 256 × 256, field of view = 240 × 240 mm^2^, slice thickness = 5 mm, and slice spacing = 1.5 mm) was collected to rule out any cranial organic lesion. Then, a sagittal three-dimensional T1-weighted fast spoiled gradient recalled echo (T1-FSPGR) sequence was acquired with the following parameters: TR = 7.8 ms, TE = 3.0 ms, matrix size = 256 × 256, field of view = 256 × 256 mm^2^, slice thickness = 1 mm, and 166 slices. Then, a gradient-echo T2^*^-weighted echo planar imaging sequence was used for acquiring functional images with the following parameters: TR = 2,000 ms, TE = 30 ms, matrix size = 64 × 64, FOV = 256 × 256 mm^2^, flip angle = 90, in-plane resolution of 4 mm^2^, slice thickness = 4 mm (with no slice gap), and 32 axial slices. The scan for RS-fMRI lasted 360 seconds, and participants were instructed to view a fixation cross and try to remain awake with their eyes open while not thinking of anything during the scanning procedure. After the resting-state scan, fMRI food cue–reactivity task was performed and lasted 390 seconds.

### Image processing.

All imaging data were analyzed using Statistical Parametric Mapping 12 (SPM12, http://www.fil.ion.ucl.ac.uk/spm). The functional images first underwent conventional slice-timing, head movement correction. The echo-planar images were coregistered to each individual’s T1 anatomical image and spatially normalized to the template of the Montreal Neurological Institute and resampled to a voxel size of 3 mm^3^. For resting-state data, demeaning and detrending were also performed, and head-motion parameters and white matter and cerebrospinal fluid (CSF) signals were regressed out as nuisance covariates. RS-fMRI time points that were severely affected by motion were scrubbed using a “scrubbing method” (framewise displacement [FD] value > 0.5 mm and ΔBOLD of DVARS > 0.5%), and less than 5% of time points were scrubbed per participant. ALFF analysis was carried out to measure spontaneous fluctuations in resting-state BOLD-fMRI signal intensity. For the cue-reactivity task imaging data, an isotropic Gaussian-kernel was used to spatially smooth the images (6 mm). The general-linear-model, including HiCal and LoCal food cue condition regressors, was constructed. Individual β images responding to HiCal versus LoCal cues were computed.

### Statistics.

A 2-way repeated-measures ANOVA was implemented in SPSS 22 to model the effects of group and time on behavioral/clinical data. Paired, 2-tailed *t* tests were utilized as post hoc tests in cases where ANOVA indicated significant main/interaction effects. Two-sample, 2-tailed *t* test was used to examine the difference between AT and TT groups. False discovery rate correction was applied for multiple comparisons, and the level of significance was set at *P* < 0.05.

SPM12 was used to perform voxel-wise analysis on resting-state activity/food cue–induced activation. Age and sex were entered as covariates to control for differences between groups in these variables. A 2-way repeated-measures ANOVA was implemented to model the group, time, and interaction effects. Results were corrected for multiple comparisons using family-wise error (FWE) correction at the cluster level (*P*_FWE_ < 0.05), with a minimum cluster size of *k* = 50 voxels and a cluster defining threshold of *P* < 0.001. The clusters with significant interaction and group effects were selected as the seed regions, and then resting-state functional connectivity and whole-brain PPI analyses were performed.

We conducted a partial correlation analysis with age and sex as covariates to assess the associations between ALFF and β values in the regions with statistical significance and clinical data, as well as correlations between changes in ALFF/β values and changes in behavioral measurements (24). False discovery rate correction was applied for multiple comparisons, and the level of significance was set at *P* < 0.05.

### Study approval.

The experimental protocol was approved by the Institutional Review Board of Xijing Hospital, and the experiments were conducted in accordance with the Declaration of Helsinki. All participants were informed of the nature of the research and provided written informed consent.

### Data availability.

Values for all data points in graphs are reported in the Supporting Data Values file. Other data from this study are available upon reasonable request.

## Author contributions

YZ and GJW designed the study. GJ performed bariatric surgery. GL, JY, LS, and JW performed data collection. GL, YH, and WZ analyzed the data. GL drafted the manuscript. PM and NDV provided critical revision of the manuscript. All authors critically reviewed the content and approved the final version for publication. GL, YH, and WZ contributed equally. GL was listed first because he drafted the manuscript and YH is listed before WZ based on alphabetical order of surname.

## Supplementary Material

Supplemental data

ICMJE disclosure forms

Supporting data values

## Figures and Tables

**Table 1:**
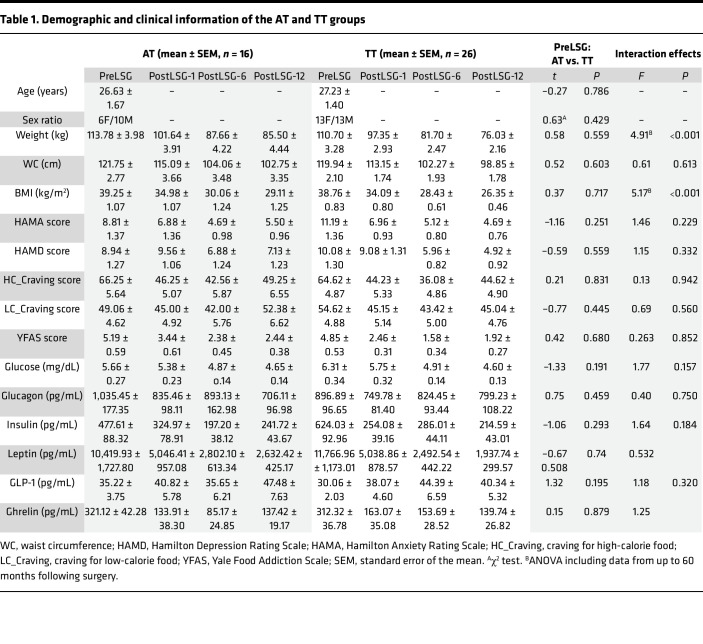
Demographic and clinical information of the AT and TT groups

**Table 2:**
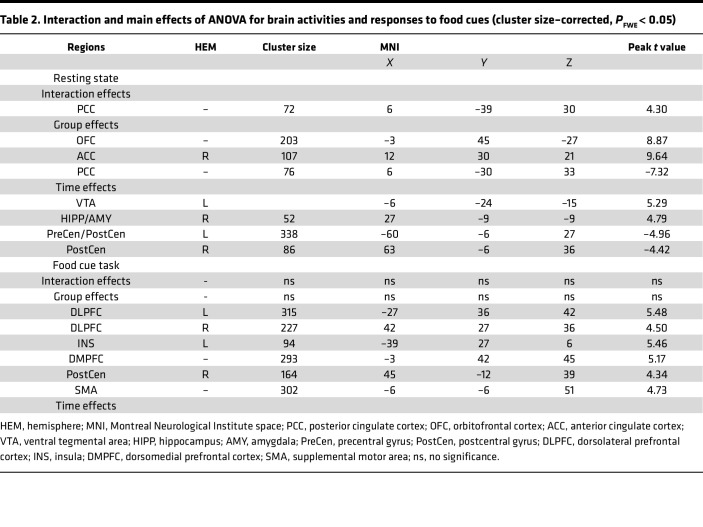
Interaction and main effects of ANOVA for brain activities and responses to food cues (cluster size–corrected, *P*_FWE_ < 0.05)

## Version 1 08/01/2024

In-Press Preview

## Version 2 09/10/2024

Electronic publication
