# Supplementary material for: FTO variant is associated with changes in BMI, ghrelin, and brain function following bariatric surgery
Source: JCI Insight. 2024 Aug 1;9(17):e175967. doi: 10.1172/jci.insight.175967 (PMC11385082; doi:10.1172/jci.insight.175967)

Supplementary Information

Materials and Methods

Peripheral hormone measurement

Blood samples were collected into EDTA-coated tubes and protease inhibitors were immediately added (10 mM DDP-IV and 1.3% Aprotinin, both dissolved with 0.9% NaCl). The protease inhibitors were prepared according to the kit manufacturer's recommendation (Bio-Plex). After mixing inhibitors and blood, samples were centrifuged at 1400 × g per 10 min at 4°C, and the supernatants were stored at -80°C before analysis.

Plasma concentrations of total ghrelin, GLP-1, glucagon, leptin and insulin were measured using a Bio-Plex 200TM suspension array system (BIO-RAD, Inc, Hercules, California, USA). The principle of the Bio-Plex Pro bead-based assays is similar to capture sandwich immunoassays. The capture antibody-coupled beads were first incubated with the sample followed by incubation with biotinylated detection antibodies. After washing away the unbound biotinylated antibodies, the beads were incubated with a reporter streptavidin-phycoerythrin conjugate. The beads were then passed through the Bio-Plex array reader, which measures the fluorescence of the bound streptavidin-phycoerythrin on each bead. Measurements were provided as the median fluorescence intensity for a given bead population. Bead populations were identified by fluorescence to determine the analyte being measured. All assay incubations were performed at room temperature as described in the instruction manual (Bio-Rad part number 10024929). All washes were performed using a Bio-

Plex Pro Wash Station with cycles of 200 μL of wash buffer per well. Data acquisition was done using the Bio-Plex Manager 6.1 software. The inter-assay variation (% coefficient of variation (CV)) was 4%, and the intra-assay variation (%CV) was 5%.

Supplementary Tables

Supplementary Table 1. Genotype analysis of SNPs of FTO and MC4-R.

**Genotype**		***FTO***				***MC4-R***	
		rs8050136: AA/AC/CC	*rs17817449:* GG/GT/TT	*rs1421085:* CC/CT/TT	*rs1121980:* TT/TC/CC	*rs17782313:* CC/CT/TT	*rs12970134:* AA/AG/GG
FTO-rs9939609	AT:16	0/11/5	0/12/4	0/11/5	0/15/1	0/6/10	0/6/10
	TT:26	0/0/26	0/0/26	0/0/26	0/0/26	0/5/21	1/6/19

Supplementary Table 2. Group comparison of BMI within FTO and MC4-R variants.

Genotype		PreLSG	PostLSG-1	PostLSG-6	PostLSG-12	PostLSG-24	PostLSG-36	PostLSG-48	PostLSG-60
FTO-rs9939609	**AT: 16**	39.25±1.07	34.98±1.07	30.06±1.24	29.11±1.25	29.30±1.24	30.27±1.19	31.23±1.19	32.53±1.17
	**TT: 26**	38.76±0.83	34.09±0.80	28.43±0.61	26.35±0.46	26.81±0.46	27.28±0.59	28.11±0.68	28.26±0.69
	**AT vs TT (t/P)**	0.36/0.717	0.67/0.504	1.31/0.197	**2.43/0.020**	**2.18/0.035**	**2.49/0.017**	**2.44/0.019**	**3.33/0.002**
FTO-rs8050136	**AC: 11**	39.87±1.47	35.41±1.53	30.80±1.73	30.02±1.68	30.13±1.72	31.03±1.64	31.91±1.64	33.09±1.60
	**CC: 31**	38.62±0.71	34.08±0.67	28.43±0.53	26.47±0.42	26.92±0.41	27.49±0.52	28.38±0.60	28.75±0.64
	**AC vs CC (t/P)**	0.84/0.402	0.91/0.365	1.75/0.087	**2.91/0.006**	**2.60/0.013**	**2.70/0.010**	**2.51/0.016**	**3.01/0.005**
FTO-rs17817449	**GT: 12**	39.51±1.39	35.21±1.41	30.49±1.61	29.53±1.61	29.84±1.60	30.69±1.54	31.63±1.52	32.71±1.51
	**TT: 30**	38.72±0.73	34.11±0.69	28.47±0.54	26.55±0.43	26.93±0.43	27.51±0.54	28.37±0.62	28.76±0.66
	**GT vs TT (t/P)**	0.53/0.594	0.77/0.445	1.51/0.138	**2.45/0.019**	**2.40/0.021**	**2.45/0.018**	**2.36/0.023**	**2.78/0.008**
FTO-rs1421085	**CT: 11**	39.87±1.47	35.41±1.53	30.80±1.73	30.02±1.68	30.13±1.72	31.03±1.64	31.91±1.64	33.09±1.60
	**TT: 31**	38.62±0.71	34.08±0.67	28.43±0.53	26.47±0.42	26.92±0.41	27.49±0.52	28.38±0.60	28.75±0.64
	**CT vs TT (t/P)**	0.84/0.402	0.91/0.365	1.75/0.087	**2.91/0.006**	**2.60/0.013**	**2.70/0.010**	**2.51/0.016**	**3.01/0.005**
FTO-rs1121980	**TC: 15**	39.28±1.14	34.99±1.14	30.24±1.30	29.43±1.28	29.56±1.30	30.54±1.24	31.44±1.25	32.77±1.23
	**CC: 27**	38.76±0.80	34.11±0.76	28.38±0.59	26.28±0.44	26.76±0.45	27.24±0.57	28.11±0.66	28.29±0.66
	**TC vs CC (t/P)**	0.37/0.707	0.65/0.515	1.48/0.146	**2.80/0.008**	**2.46/0.018**	**2.75/0.009**	**2.59/0.013**	**3.49/0.001**
MC4-R-rs17782313	**CT: 11**	40.20±1.38	35.74±1.30	30.29±1.70	28.96±1.59	29.49±1.40	30.02±1.48	30.97±1.52	31.44±1.69
	**TT: 31**	38.50±0.72	33.96±0.72	28.61±0.56	26.85±0.53	27.15±0.58	27.85±0.63	28.71±0.69	29.34±0.71
	**CT vs TT (t/P)**	1.15/0.257	1.24/0.222	1.22/0.229	1.63/0.111	1.83/0.074	1.56/0.125	1.54/0.131	1.34/0.187
MC4-R-rs12970134	**AA/AG: 13**	39.58±1.13	35.33±1.11	30.05±1.42	28.57±1.33	28.94±1.17	29.63±1.24	30.17±1.29	30.73±1.44
	**GG: 29**	38.66±0.80	34.02±0.77	28.60±0.61	26.88±0.58	27.23±0.64	27.88±0.69	28.91±0.75	29.51±0.77
	**AA/AG vs TT (t/P)**	0.64/0.520	0.94/0.350	1.10/0.276	1.35/0.182	1.37/0.176	1.32/0.194	0.88/0.381	0.80/0.425

Supplementary Table 3. Time effects and post-hoc tests for behavioral measurements.

	Time effects		Post-hoc tests	PreLSG vs PostLSG-1		PostLSG-1 vs PostLSG-6		PostLSG-6 vs PostLSG-12		PreLSG vs PostLSG-12	
	F	*P*		t	*P*	t	*P*	t	*P*	t	*P*
**Weight (kg)**	370.81	< 0.001	AT	12.62	<0.001	11.38	<0.001	2.34	0.033	15.65	<0.001
			TT	17.117	<0.001	12.463	<0.001	6.367	<0.001	17.80	<0.001
**WC (cm)**	202.08	< 0.001	AT	3.53	0.003	7.86	<0.001	3.02	0.009	14.12	<0.001
			TT	6.298	<0.001	10.684	<0.001	8.234	<0.001	13.57	<0.001
**BMI**	399.38	< 0.001	AT	13.88	<0.001	9.93	<0.001	3.32	0.005	9.82	<0.001
			TT	19.9	<0.001	12.28	<0.001	6.62	<0.001	13.04	<0.001
**HAMA**	18.05	< 0.001	AT	1.992	0.065	2.22	0.042	-1.46	0.165	1.42	0.177
			TT	3.28	0.003	2.78	0.010	0.66	0.515	2.81	0.009
**HAMD**	8.3	< 0.001	AT	-0.57	0.575	2.17	0.046	-0.25	0.809	1.78	0.095
			TT	0.63	0.534	2.73	0.011	2.11	0.045	3.94	0.001
**HiCal Craving**	12.63	< 0.001	AT	3.09	0.007	0.82	0.425	-1.15	0.269	-0.46	0.65
			TT	2.96	0.007	1.24	0.225	-2.57	0.016	-0.07	0.94
**LoCal Craving**	1.54	0.208	AT	0.78	0.449	0.47	0.642	-1.55	0.142	-1.07	0.3
			TT	1.46	0.158	0.31	0.755	-0.4	0.693	0.02	0.983
**YFAS**	25.19	< 0.001	AT	3.36	0.004	1.47	0.162	-0.14	0.889	1.62	0.127
			TT	3.53	0.002	2.89	0.008	-1.51	0.142	1.68	0.105
**Glucose**	26.23	< 0.001	AT	3.57	0.003	2.50	0.024	2.76	0.015	3.47	0.003
			TT	4.22	<0.001	3.46	0.002	4.33	<0.001	5.48	<0.001
**Glucagon**	1.41	0.244	AT	1.64	0.122	-0.29	0.775	1.21	0.246	1.38	0.186
			TT	1.07	0.293	-1.09	0.285	0.25	0.806	0.57	0.571
**Insulin**	14.81	< 0.001	AT	1.31	0.208	1.31	0.208	-1.16	0.263	3.22	0.006
			TT	3.95	<0.001	-0.71	0.480	2.21	0.037	4.49	<0.001
**Leptin**	61.95	< 0.001	AT	3.89	0.001	2.27	0.038	0.25	0.806	4.67	<0.001
			TT	9.58	<0.001	3.48	0.002	1.41	0.172	8.80	<0.001
**GLP-1**	2.05	0.110	AT	-0.98	0.339	0.81	0.426	-1.34	0.20	-1.25	0.23
			TT	-2.13	0.043	-1.18	0.248	0.95	0.350	-1.88	0.071
**Ghrelin**	43.02	< 0.001	AT	4.21	<0.001	1.93	0.073	-4.92	<0.001	4.36	<0.001
			TT	6.56	<0.001	0.37	0.714	0.66	0.512	5.77	<0.001

Supplementary Table 4. Group effects and post-hoc tests for behavioral measurements.

	Group effects		PreLSG: AT vs. TT		PostLSG-1: AT vs. TT		PostLSG-6: AT vs. TT		PostLSG-12: AT vs. TT	
	F	P	t	*P*	t	*P*	t	*P*	t	*P*
**Weight (kg)**	0.76	0.389	0.59	0.559	0.89	0.380	1.30	0.200	2.14	0.039
**WC (cm)**	0.49	0.486	0.53	0.603	0.54	0.594	0.49	0.627	1.13	0.267
**BMI**	1.46	0.235	0.37	0.717	0.67	0.504	1.31	0.197	2.38	0.022
**HAMA**	0.18	0.676	-1.16	0.251	-0.05	0.956	-0.34	0.739	0.66	0.514
**HAMD**	0.23	0.635	-0.59	0.559	0.26	0.796	0.64	0.527	1.456	0.154
**HiCal Craving**	0.44	0.512	0.21	0.831	0.26	0.799	0.84	0.406	0.576	0.5694
**LoCal Craving**	<0.01	0.992	-0.77	0.445	-0.02	0.984	-0.18	0.857	0.92	0.364
**YFAS**	2.66	0.111	0.42	0.68	1.57	0.123	1.44	0.158	1.15	0.258
**Glucose**	0.72	0.401	-1.33	0.191	-0.82	0.414	-0.18	0.852	0.24	0.805
**Glucagon**	0.31	0.581	0.74	0.459	0.66	0.511	0.39	0.696	-0.59	0.559
**Insulin**	0.32	0.578	-1.06	0.293	0.89	0.377	-1.39	0.172	0.41	0.677
**Leptin**	0.01	0.929	-0.66	0.508	0.01	0.996	0.41	0.678	1.37	0.178
**GLP-1**	0.08	0.773	1.31	0.195	0.37	0.713	-0.89	0.374	0.79	0.435
**Ghrelin**	0.31	0.579	0.15	0.879	-0.54	0.592	-1.66	0.105	-0.06	0.954

Supplementary Table 5. Group effects of ANOVA for resting state functional connectivity and PPI connectivity in responses to food cues.

Functional Connectivity	HEM	Cluster Size	MNI			Peak T-value
			X	Y	Z	
Resting State: Group effects						
PCC-DLPFC	L	136	-21	48	33	-5.97
PCC-ANG	L	191	-45	-66	48	-10.61
Food Cue Task: Group effects						
DLPFC- Hippocampus	L	91	-27	-30	-9	4.42
DMPFC- Caudate	L	85	-18	9	12	3.95

Abbreviation: PCC, posterior cingulate cortex; DLPFC, dorsolateral prefrontal cortex; ANG, angular gyrus; DMPFC, dorsomedial prefrontal cortex.

Supplementary Figure

Supplementary Figure 1. The correlation between basal BMI and BMI reductions at, 24-, 36-, 48- and 60- months after LSG in the TT group.

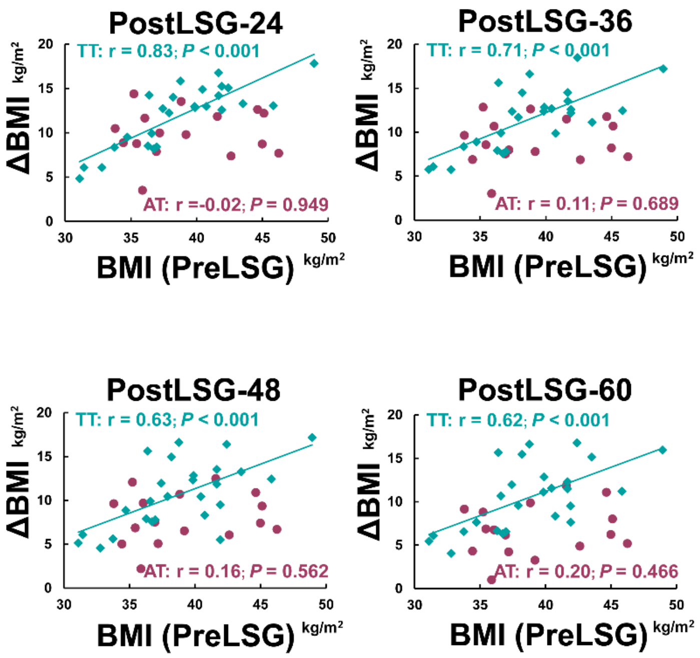

Supplementary Figure 2. The correlation between the rebounding ghrelin at PostLSG-12 and BMI at, 24-, 36-, 48- and 60- months after LSG in the AT group.

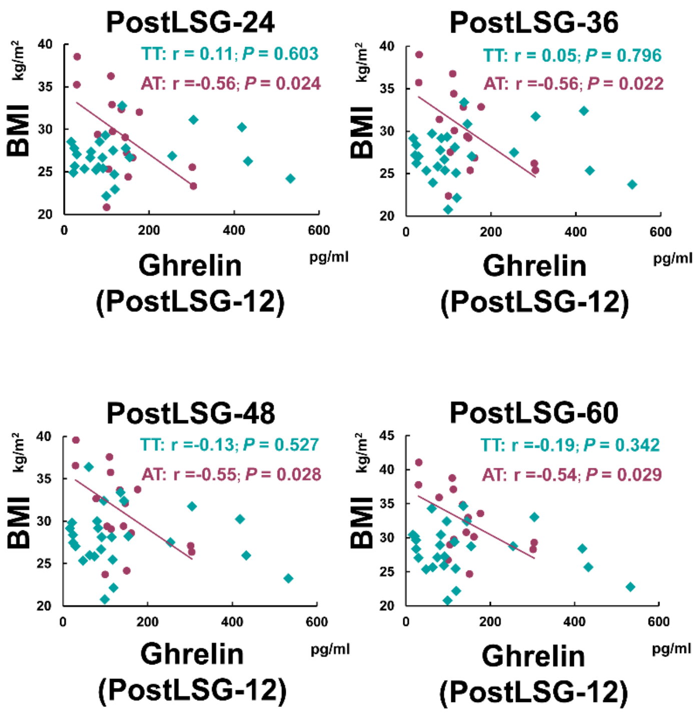

Supplementary Figure 3. Time effects of ANOVA for HiCal food craving. In the AT group, HiCal food craving at PostLSG-12 was negatively correlated with the EBMIL at 12-, 24-, 36-, 48- and 60- months after LSG.

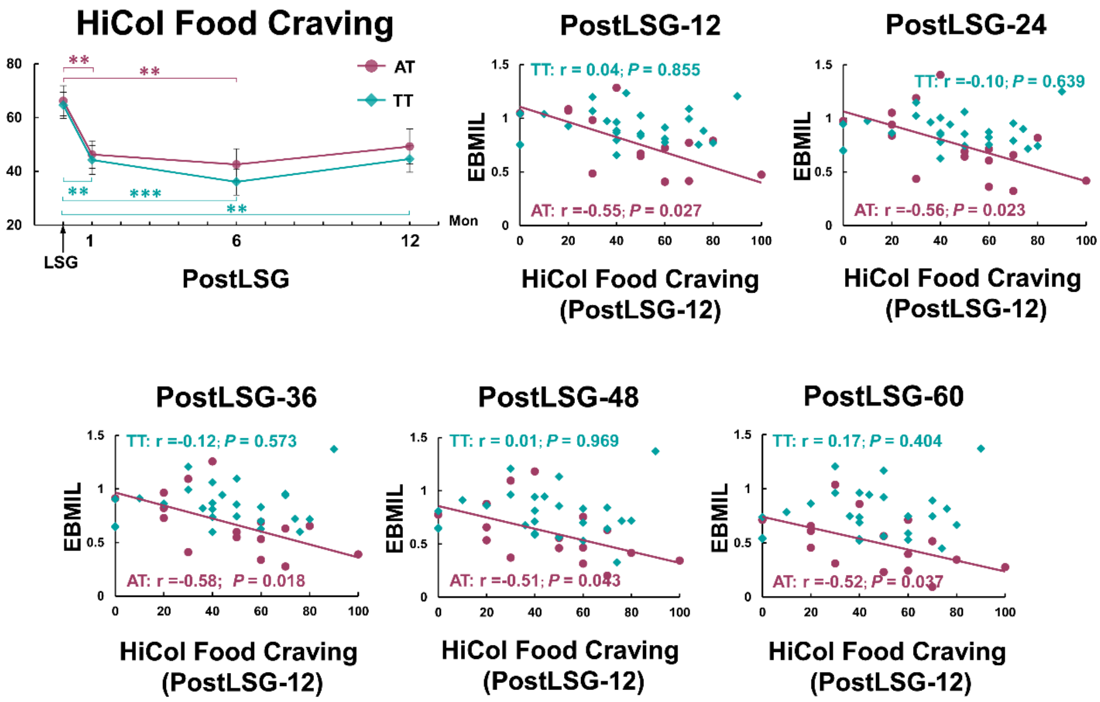

Supplementary Figure 4. Group effects of ANOVA for resting state functional connectivity and PPI connectivity in responses to food cues.

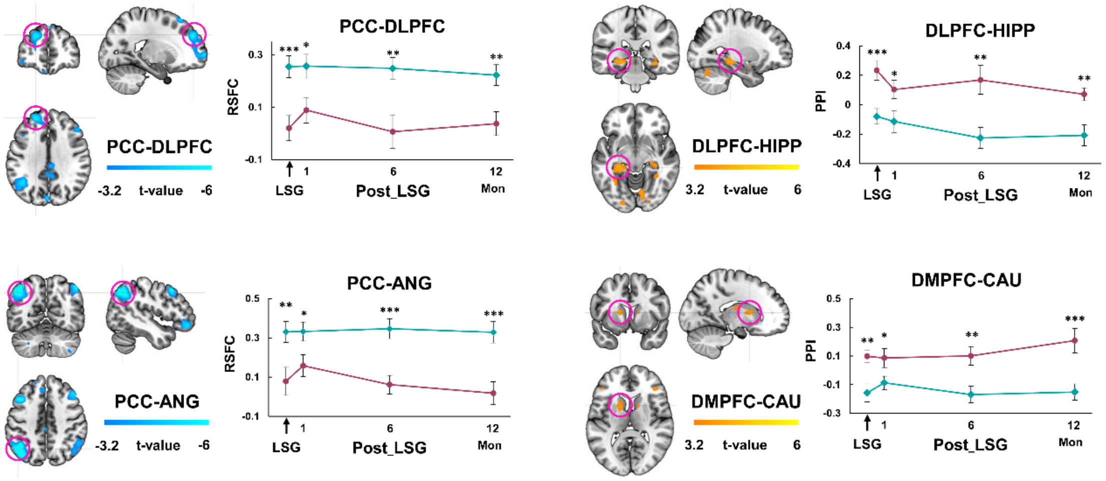

Supplement: Supplemental data [file jciinsight-9-175967-s226.pdf]
